# Inhibitory Effect of Aqueous Extracts from Marine Sponges on the Activity and Expression of Gelatinases A (MMP-2) and B (MMP-9) in Rat Astrocyte Cultures

**DOI:** 10.1371/journal.pone.0129322

**Published:** 2015-06-08

**Authors:** Gaetano Di Bari, Eugenia Gentile, Tiziana Latronico, Giuseppe Corriero, Anna Fasano, Carlotta Nonnis Marzano, Grazia Maria Liuzzi

**Affiliations:** 1 Department of Biosciences, Biotechnologies and Biopharmaceutics, Aldo Moro University, Bari, Italy; 2 Department of Biology, Aldo Moro University, Bari, Italy; National Center for Scientific Research Demokritos, GREECE

## Abstract

The aim of this study was to evaluate whether water soluble compounds present in aqueous extracts from seven Mediterranean demosponges exert biological activity towards matrix metalloproteinases (MMPs), which represent important pathogenic factors of human diseases. Aqueous extracts were tested on LPS-activated cultured rat astrocytes, and levels and expression of MMP-2 and MMP-9 were assessed by zymography and RT-PCR, respectively. Our results demonstrated that the studied extracts contain water soluble compounds able to inhibit MMP-2 and MMP-9 activity and expression. We also compared the anti-MMP activities present in aqueous extracts from wild and reared specimens of *Tethya aurantium* and *T*. *citrina*. The results obtained revealed that the reared sponges maintain the production of bioactive compounds with inhibitory effect on MMP-2 and MMP-9 for all the duration of the rearing period. Taken together, our results indicate that the aqueous extracts from the selected Mediterranean demosponges possess a variety of water-soluble bioactive compounds, which are able to inhibit MMPs at different levels. The presence of biological activity in aqueous extracts from reared specimens of *T*. *aurantium* and *T*. *citrina* strongly encourage sponge aquaculture as a valid option to supply sponge biomass for drug development on a large scale.

## Introduction

Matrix metalloproteinases (MMPs) are a family of zinc-dependent, neutral endopeptidases which degrade the components of the extracellular matrix (ECM), such as collagen, fibronectin, elastin and laminin [1,2]. In physiological conditions, their expression is tightly regulated by growth factors, cytokines and steroids, whereas their activity is controlled by their endogenous inhibitors, the tissue inhibitors of metalloproteinases (TIMPs) that bind to the latent forms of MMP inactivating the enzyme. The over-expression of MMPs by various cell types has been implicated in the pathogenesis of many diseases and diverse invasive processes, including arthritis, cardiovascular diseases, stroke, multiple sclerosis, neurodegenerative diseases, allergies as well as cancer [3–4]. Several experimental evidence indicate the involvement of gelatinases A (MMP-2) and B (MMP-9) in the pathogenesis of HIV-associated dementia (HAD) [5], Multiple Sclerosis (MS) [6–8] and cancer progression [9]. Therefore, these enzymes have been considered therapeutic targets for the treatment of the mentioned diseases. In this respect, in the past decade different synthetic inhibitors of MMPs have been used in clinical trials for cancer and other diseases [10,11], but this approach gave equivocal results in terms of selectivity and efficacy [12,13]. Therefore, the research has been oriented at evaluating whether the drugs, currently used for the treatment of diseases in which MMP are involved, could exert their beneficial effect also by reducing the levels and expression of these enzymes. In this respect, it has been demonstrated that drugs used for the treatment of MS patients (e.g. IFN-β) and HIV-infected patients (e.g. zidovudine, indinavir and maraviroc) are able to inhibit the activity and expression of MMP-2 and MMP-9 [14–17]. In recent years, the attention has been focused on the identification of natural products with anti-MMP properties. In this respect, we have shown that natural antioxidant compounds and omega-3 polyunsaturated fatty acids, extracted from fish oil, are able to inhibit the activity and expression of MMP-2 and MMP-9 in rat glial cells [18,19]. Present approaches in the research of natural compounds with anti-MMP activities are addressed to the search of bioactive compounds isolated from marine organisms. Indeed, different compounds with anti-MMP activities have been purified from marine sponges [20], which represent a promising source of bioactive compounds with interesting pharmacological activities against a variety of human diseases [21,22]. Specifically, until now the anti-MMP compounds isolated from marine sponges are mostly represented by lipophilic organic molecules such as ageladine A [23], ancorinosides B-D [24] and aeroplysinin-1 [25], which are able to exert their anti-MMP inhibitory activity with high selectivity. However, so far the biological potential of water-soluble compounds, and in particular of proteins, isolated from marine sponges, has been little studied. Therefore, it would be very interesting to evaluate whether water-soluble compounds extracted from marine sponges possess anti-MMP properties. In this respect, we have recently reported the preparation of aqueous extracts from seven Mediterranean demosponges, *Tethya aurantium*, *T*. *citrina*, *Hymeniacidon perlevis*, *Ircinia variabilis*, *Chondrilla nucula*, *Aplysina aerophoba* and *Sarcotragus spinosulus*. The analysis on sodium dodecyl sulphate polyacrylamide electrophoresis (SDS-PAGE) evidenced the presence of specific protein profiles in the different sponge extracts. In addition, we determined the non-cytotoxic concentrations of each extract in four mammalian cell types [26].

On these grounds, the purpose of this study was to determine whether the aqueous extracts, prepared from the above mentioned Mediterranean demosponges, possess inhibitory activity against gelatinases A (MMP-2) and B (MMP-9). To this end, we used an *in vitro* model already used in our laboratory [27], represented by rat astrocytes stimulated with LPS, a well-know inducer of MMPs [28]. We demonstrated that the aqueous extracts are able to exert inhibitory effect on MMP activity and expression. In addition, in our research we showed that the observed inhibitory activity against MMPs was maintained also in the aqueous extracts prepared from reared specimens of the two Mediterranean species, *T*. *aurantium* and *T*. *citrina*.

## Materials and Methods

### Ethics Statement

No specific permits were required for the described field studies. The sponge samples were collected from a location that is not privately-owned or protected in any way, according to the authorization of Marina Mercantile now called Ministero delle Politiche Agricole, Alimentari e Forestali (DPR 1639/68, 09/19/ 1980 confirmed on 01/10/2000). The Department of Biology of Universityof Bari has been approved by the Ministero della Marina Mercantile as an “Accredited Scientific Institution” and it is in compliance with all formalities required from “Accredited Scientific Institutions”, as reported in the Art. 27, 29, 30 of the DPR 1639/68. The field studies did not involve endangered or protected species. All animal procedures were in compliance with the guidelines of the European Union (directive 609/86).

All experimental procedures involving animals were carried out in strict accordance with the recommendations in the NIH Guide for the Care and Use of Laboratory Animals and approved by the Institutional Animal Care and Use Committee of University of Bari, Italy (Permit Number: 23-98-A). All efforts were made to minimize the number of animals used and to ameliorate their suffering.

### Chemicals

Dulbecco’s modified Eagle’s medium (DMEM), fetal bovine serum (FBS), penicillin and streptomycin, L-glutamine were obtained from GIBCO (Paisley, Scotland). DNase 1, gelatin, lypopolysaccharide (LPS), 1,10 phenanthroline (PA), poly-L-lysine (PLL), trypsin, trypan blue, 3-(4,5-dimethylthiazol-2-yl)-2,5 diphenyltetrazolium bromide (MTT), phorbol 12-myristate 13-acetate, bovine serum albumine was provided by Sigma (St. Louis, MO, USA). Glial fibrillary acidic protein (GFAP) antibodies were purchased from Serotec (Oxford, UK). Standard proteins, G-250and R-250 coomassie brilliant blue were purchased from Bio-Rad (Hercules, CA, USA). Purified MMP-2 and MMP-9 were purchased from Alexis Biochemicals (San Diego, CA, USA). Primer pairs specific for MMP-2, MMP-9 and 18S were from Sigma Genosys (Cambridge, UK). RNeasy mini kit and QuantiTect Reverse Transcription were from Qiagen (Valencia, CA, USA). Antibodies against extracellular–regulated protein kinases (ERK) 1/2, and phosporilated ERK 1/2 (p-ERK 1/2) were from Santa Cruz Biotechnology (Santa Cruz, CA). Hybond-P PVDF membranes, enhanced chemiluminescence (ECL) Western Blotting Analysis System and anti-mouse-HRP secondary antibody were from GE Healthcare Life Sciences (Little Chalfont, Buckinghamshire, UK).

### Sponge collection

For our experiments seven different demosponges, commonly found in the Adriatic Sea (Palese, Bari; N 41°09’ 39” E 16°45’ 50”), were collected. In particular, specimens from *T*. *aurantium*, *T*. *citrina*, *H*. *perlevis*, *I*. *variabilis*, *C*. *nucula*, *A*. *aerophoba* and *S*. *spinosulus* were chosen.

Sponge species were collected by scuba diving in Southern Adriatic Sea, Italy, at depths between 1 and 3 meters. Sponges were individually transferred to laboratory in bags filled with seawater and labelled for recognition. During the transport, the samples were protected against contact with air as well as other injuries and temperature was maintained around 18°C. Once in laboratory, all sponges were numbered and listed with information like date of sampling and location, weighed and then frozen at -80°C as soon as possible until the extraction.

### *Tethya aurantium* and *T*. *citrina* collection and maintenance in aquarium

A total of 92 specimens of *T*. *aurantium* and 74 of *T*. *citrina* were collected from the Adriatic Sea (Palese, Bari; N 41°09’ 39” E 16°45’ 50”), on rocky bottom, around 2 meters of depth, from February to March 2010. The sponges were placed in closed tanks containing seawater, transported to the laboratory, tagged, photographed [29] and then reared in three 150 l volume tanks. Each tank was filled with natural seawater maintained at a temperature and salinity of 21 (±1.2)°C and 37 (±0.6) psu, respectively. The stocking density was approximately 1 specimen per 0.6 l of water. The filtering system used was a mechanical coarse. A replacement of 10% of total water was daily carried out, using seawater from the sampling site, and, in addition, sponge diet was daily supplemented with 300 cc of solution containing the microalga *Nannochloropsis sp*. (2 μm diameter) at a concentration of about 4×10^7^ cells/cc. Taking into account the different number of the specimens of the studied species, aqueous extracts were prepared throughout a period of 18 months for *T*. *aurantium* and of 12 months for *T*. *citrina*.

### Preparation of aqueous extracts

Sponges were homogenized in extraction buffer, consisting of 10 mM sterile phosphate buffered saline (PBS), 150 mM NaCl, pH 7.0 (1:4 w/v), according the methodology proposed by [26]. Specimens from *T*. *aurantium* and *T*. *citrina* were grounded in phosphate buffered saline with mortar and pestle set in an iced bath. The other sponges were, instead, homogenized in the same buffer in a waring blender. The homogenate was subjected to three cycles of freezing (20 min, -20°C) and thawing (10 min, RT). To avoid any microbial contamination due to the symbiotic bacteria, penicillin/streptomycin (5x10^3^ U/ml) was added to the homogenate, which was then centrifuged for 60 minutes at 15.000 g, 4°C. The clear supernatant was collected and filtered through a Millipore membrane, pore size 0.22μm. Total protein content of each extract was determined according to Bradford method [30]. Extracts were then kept at -80°C until further analysis.

In order to exclude the presence of residual organic metabolites in the sponge extracts, amounts of crude extract from the different sponges, corresponding to 500 μg of proteins, were subjected to a further step of extraction with two volumes of chloroform (CHCl_3_). After centrifugation for 3 minutes at 13000 g a biphasic separation, consisting of a top aqueous (aqueous phase) and a bottom organic phase, was obtained. The two fractions were collected separately and the CHCl_3_ phase was dried by nitrogenous stream and then resuspended in CHCl_3_/PBS (1:10 v/v) (organic phase). Protein concentration was measured by using the Bradford assay in both the aqueous and the organic phases, then samples were kept at -80°C until further testing on astrocytes.

### Preparation of astrocyte primary cultures

One-day old rats were anesthetized with ether vapors, then sacrificed by rapid decapitation and the dissected neocortical tissues were used for the preparation of primary glial cell cultures as described by [31]. Astrocytes were then purified from the mixed culture by trypsinization and replating to remove contaminating microglia and oligodendrocytes. As assessed by immunostaining for GFAP more than 98% of the cells were GFAP-positive in all the preparations. Astrocytes were plated in PLL-coated 96-well plates at a density of 1x10^5^ cells/ml (100 μl/well) in complete medium (DMEM supplemented with 100 U/ml penicillin, 100 μg/ml streptomycin, 10% fetal bovine serum) and maintained at 37°C in a 5% CO_2_.

### Treatment of astrocytes with LPS and aqueous extracts

One day after seeding at a density of 1 x 10^5^ cells/ml in 96-well plates, astrocytes were washed once with serum-free DMEM, activated with LPS at the final concentrations of 10 μg/ml and simultaneously treated with the aqueous extracts of the studied sponges at the non-cytotoxic concentrations. In another set of experiments LPS-activated astrocytes were treated with equal amounts of the aqueous extracts as well as with the corresponding aqueous and organic phases. Cells incubated in the same experimental conditions in serum-free medium served as negative controls. Positive controls were obtained from astrocytes stimulated with LPS. The treatments were performed in 100 μl of serum-free medium for 24 h at 37°C, 5% CO_2_. After incubation, the medium was collected and stored at -20°C until use. Cells were subjected to RNA extraction and polymerase chain reaction (PCR) experiments to assess MMP expression levels.

### Detection of Cytotoxicity

Cytotoxicity of astrocytes after treatment by sponge extracts was detected using the MTT assay as described in [17]. Briefly, triplicate samples of confluent astrocytes, plated in 96-well plates in serum-free DMEM, were treated for 24 h with the different sponge extracts at the final protein concentrations of 1, 5, 10, 30, 60 and 100 μg/ml. The negative control was represented by cells incubated, in the same experimental conditions, in serum-free DMEM. The difference between the absorbance of each sample at 560 and 690 nm was measured, and the value of the untreated sample was set at 100%.

### Detection of gelatinases by zymography

Gelatinases in cell culture supernatants were determined by SDS-PAGE zymography according to a modification of the method of Heussen and Dowdle [32] as described by [14]. Briefly, 50 μl of culture supernatant from astrocytes were mixed with 30 μl of SDS-loading buffer and separated in 7.5% polyacrylamide gels copolymerized with 0.1% (w/v) gelatin. Stacking gels contained 5.4% polyacrylamide. After electrophoresis, the gels were washed in 2.5% (w/v) Triton X-100, 10 mM CaCl_2_, 50 mMTris-HCl, pH 7.4 and then incubated overnight at 37°C in 1% (w/v) Triton X-100, 50 mM Tris-HCl, 10 mM CaCl_2_, pH 7.4 (developing buffer). After staining and destaining of the gels, gelatinase activity was detected as a lysis area on the blue background of the gel and was quantified by scanning densitometry and computerized image analysis using the Image Master 1D program (Pharmacia Biotech, Uppsala, Sweden).

### Reverse Transcription-polymerase chain reaction (RT-PCR)

MMP-2 and MMP-9 mRNA expression in astrocytes were determined by RT-PCR as described in [17]. Total cellular RNA was extracted from astrocytes using the Qiagen RNeasy mini kit according to the manufacturer's instructions. 500 ng RNA were used to synthesize complementary DNA (cDNA) by using the QuantiTect Reverse Transcription kit according to manufacturer’s instructions. PCR amplification was performed for MMP-2 and MMP-9 using the following primers: sense 5′-GTC ACT CCG CTG CGC TTT TCT CG-3′; antisense 5′-GAC ACA TGG GGC ACC TTC TGA-3′ for the rat MMP-2 sequence and sense 5′-CGG AGC ACG GGG ACG GGT ATC 3′; antisense 5′-AAG ACG AAG GGG AAG ACG CAC ATC 3′ for the rat MMP-9 sequence. Amplification of a 308 bp fragment of rat 18 S (sense 5’-TCCCTC AAG ATT GTC AGC AA-3’; antisense 5’-AGA TCC ACA ACG GAT ACA TT-3’), a relatively invariant internal reference RNA, was performed in parallel. Twenty-five cycles of PCR were carried out, each one consisting of denaturation at 94°C, annealing at 59°C and extension at 72°C in a thermal cycler (PTC- 100 Programmable Thermac Controller, MJ Research, Inc., Walthan, MA, USA). PCR products were visualized by ethidium bromide staining in 1.5% agarose gels. Gels were then processed for densitometric analysis as described for protein gels. Amplification of the target cDNAs were normalized to 18S expression.

### Detection of ERK-1/2 phosphorylation by Western blot analysis

ERK 1/2 was detected by immunoblot analysis as reported in [31]. Briefly, primary astrocytes plated in 6-well plates, made quiescent in serum-free medium for 24 h, were pretreated for 1 h with the crude extracts from the different sponges at the highest non-cytotoxic concentration or with 10 μM of the ERK inhibitor PD98059, then activated for 2 h with 10 μg/mg of LPS. Unstimulated and untreated astrocytes or LPS-activated astrocytes represented the negative and positive controls, respectively. After the incubation period, cells were lysed with 20mM Tris-HCl, 150mM NaCl, 2.5mM Na pyrophosphate, 1mM β-glycerophosphate, 1% Triton X-100, 1mM PMSF, 20 μg/ml aprotinin, 1mM EGTA, 1mM Na fluoride, 1mM Na3VO4, pH 7.5. Cell lysates were centrifuged at 13,000 g for 10 min at 4°C, and protein concentration was determined using the Bradford assay. Equal amounts of lysates corresponding to 60 μg of total proteins were resuspended in SDS buffer and the samples were separeted using 10% SDS-PAGE. Proteins were then immunoblotted onto PVDF membranes. After blocking overnight at 4°C with 0.05% Tween 20, 1% milk, 1% BSA in 150 mM NaCl, 20 mM Tris-HCl (TBS) (pH 7.5), blots were probed overnight at 4°C with a monoclonal anti-p-ERK-1/2 antibody (1:500). The blots were washed three times with 0.05% Tween 20 in TBS then the membranes were probed with anti-mouse-HRP secondary antibody (1:20,000) for 2 h at room temperature and detected with ECL. After exposure to autoradiographic films, membranes were stripped and incubated with an anti-rabbit antibody specific for non-phosphorylated ERK-1/2 (1:500), to assess the total amount of ERK. The quantification of levels of phosphorylation, obtained after densitometric scanning of blots and normalization to non-phosphorylated ERK-1/2, was expressed as percent compared with positive control indicated as 100%.

### Statistical analysis

The data are expressed as mean values ± standard deviation (±SD). One-way analysis of variance (ANOVA) followed by the multiple comparison Tukey test was used to assess the statistical significance between treated and untreated groups in at least three experiments with different culture preparations. A level of p< 0.05 was considered statistically significant.

## Results

### Aqueous extracts from Mediterranean sponges reduce MMP-2 and MMP-9 levels in LPS-activated astrocytes

MMP-2 and MMP-9 levels were detected by gelatin-zymography in cell culture supernatants from LPS-activated astrocytes after treatment with the aqueous extracts.

**Fig 1:**
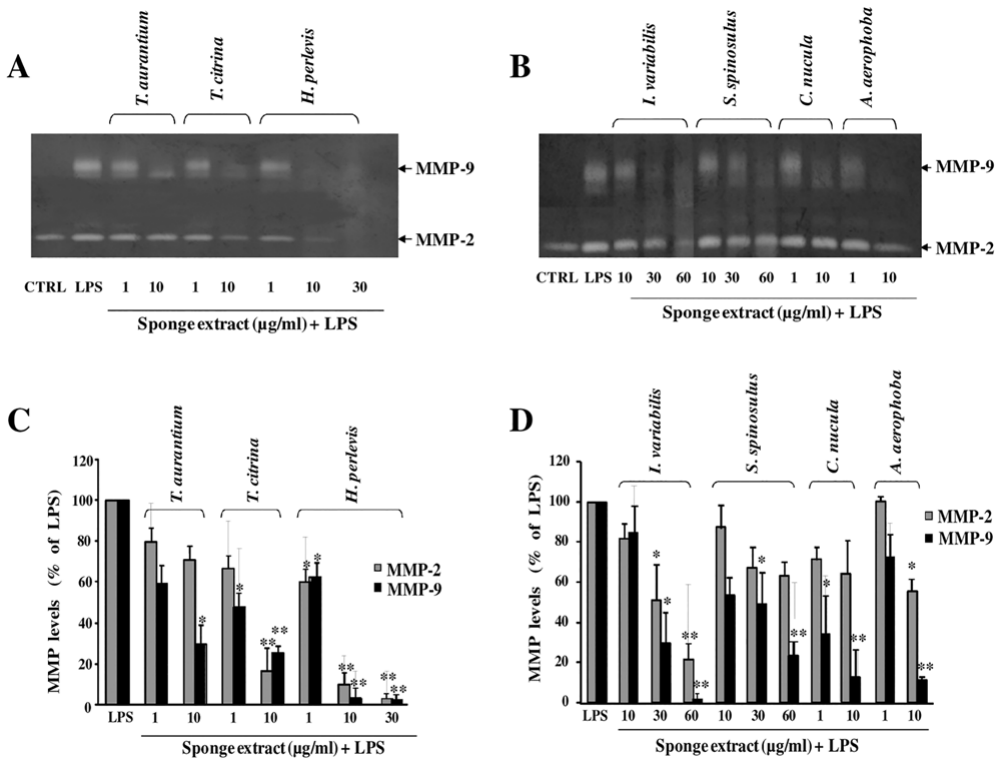
Effect of treatment with the different sponge extracts on MMP-2 and MMP-9 levels in astrocyte supernatants. Primary astrocytes (1×10^5^ cells/ml), were activated with LPS (10 μg/ml) and simultaneously treated with each sponge extract at the indicated concentrations. Untreated and unstimulated cells represent negative control (CTRL). Culture supernatants were harvested after 24 h of incubation at 37°C, 5% CO_2_ and subjected to gelatin-zymography. Representative gels in A and B show MMP-2 and MMP-9, as identified by their apparent molecular mass of 67 and 92 kDa, respectively, using pre-stained molecular weight markers (Bio Rad). Histograms in C and D represent results, expressed as mean ± SD, after scanning densitometry and computerized analysis of gels from at least three independent experiments with different cell populations. Asterisks represent values statistically different from LPS-activated astrocytes (positive control) (One-way Anova followed by Tukey test; * = p<0.05; ** p = 0.001).

As shown in the representative gels in Fig 1A and 1B, only a lysis band of 67 kDa, corresponding to MMP-2, was observed in cell culture supernatants from untreated and unstimulated astrocytes (CTRL). The treatment of astrocytes with LPS consistently induced levels of the 92 kDa MMP-9 and increased MMP-2. By contrast, the treatment of astrocytes with the sponge extracts inhibited in a dose-dependent manner MMP-2 and MMP-9 levels in LPS-activated astrocytes. In general, the changes of MMP-2 and MMP-9 levels in multiple experiments with different cell populations are shown in the histograms in Fig 1C and 1D. In particular, a statistically significant inhibition of MMP-9 and MMP-2 was observed in cells treated with the extracts from *T*. *citrina*, *H*. *perlevis*, *I*. *variabilis*, *A*. *aerophoba* at the highest concentration used. Conversely, the crude extracts from *T*. *aurantium*, *S*. *spinosulus* and *C*. *nucula*, at the highest concentration used, inhibited significantly only MMP-9.

**Fig 2:**
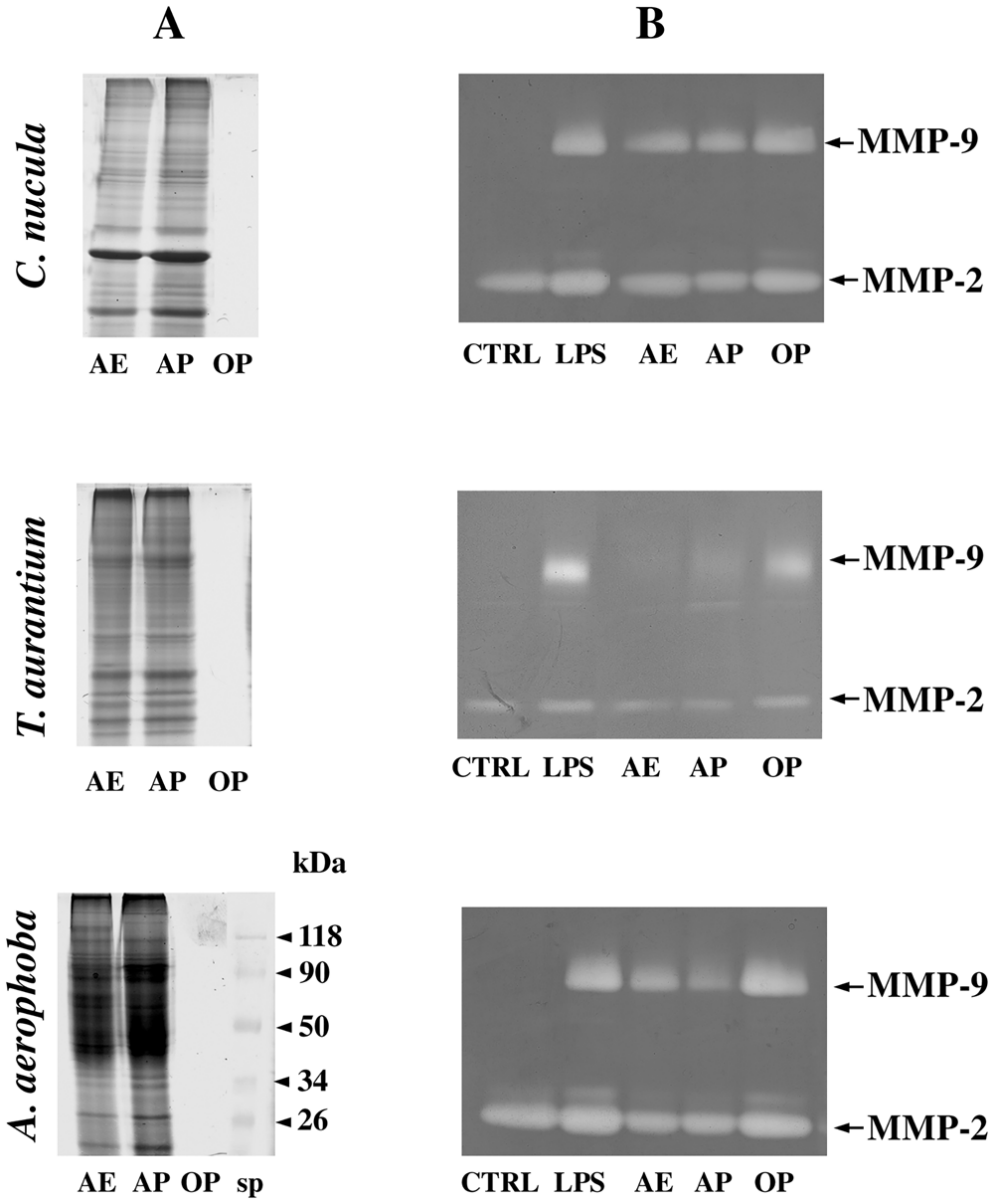
Comparison between the protein profile of the aqueous extracts and the corresponding aqueous and organic phases from the sponges *C*. *nucula*, *T*. *aurantium* and *A*. *aerophoba* and their effect on the release of MMPs from astrocytes. **A**: Thirty μg of total proteins of the aqueous extract (AE) and aqueous phase (AP) and a corresponding equal volume of organic phase (OP) were subjected to SDS-PAGE on 10% polyacrilamide electophoretic gels. Molecular weight markers (sp) (BioRad) are indicated. **B:** Representative zymographic gels of supernatants from astrocytes activated with LPS (10 μg/ml) and simultaneously treated with the crude extracts from *C*. *nucula* (10 μg/ml), *T*. *aurantium* (10 μg/ml), *A*. *aerophoba* (10 μg/ml) or with equal amounts of the corresponding aqueous and the organic phases. Untreated and unstimulated cells represent negative control (CTRL).

To ascertain that the inhibition of MMPs was not due to organic metabolites possibly present in the aqueous extracts, in another set of experiments astrocytes were treated with both the aqueous and the organic phases obtained after treatment with CHCl_3_. As shown in Fig 2A, the SDS gel electrophoresis demostrated that all the proteins present in the aqueous extracts (AE) from *C*. *nucula*, *T*. *aurantium* and *A*. *aerophoba* were still found in the aqueous phase (AP). By contrast, no proteins were found in the organic phase (OP). As observed in the rapresentative gels in Fig 2B an inhibition of MMP-2 and MMP-9, comparable with that found in supernatants from astrocytes treated with the aqueous extracts, was still observed in the supernatants from astrocytes treated with the aqueous phase from the analyzed sponges. By contrast, no inhibition was observed in supernatants from astrocytes treated with the organic phase. Similar results were obtained for all the sponges studied.

### Cytotoxicity of astrocytes after treatment with sponge extracts

**Fig 3:**
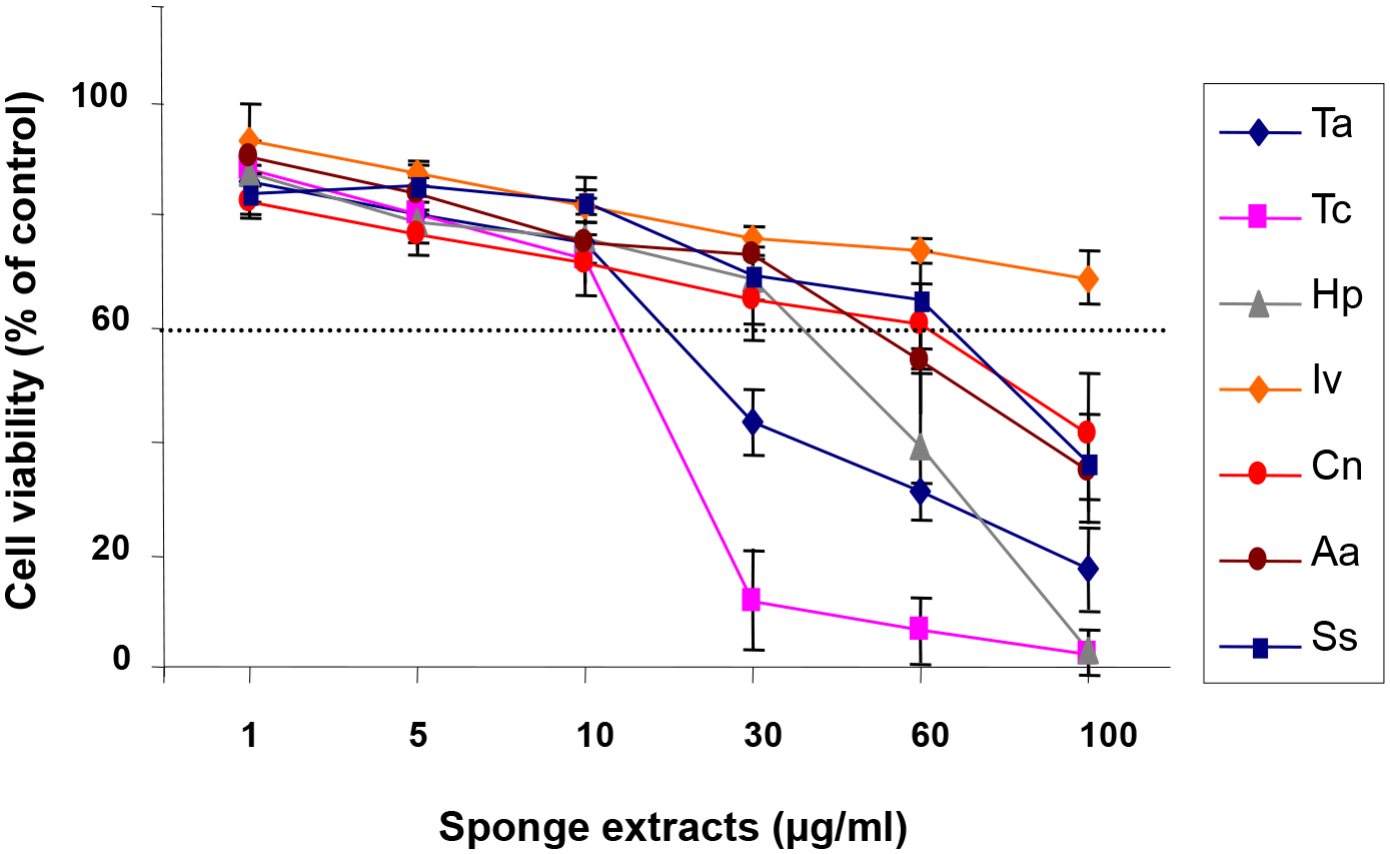
Effect of crude extracts on cell viability. Primary astrocytes (1×10^5^ cells/ml), were activated with LPS (10 μg/ml) and simultaneously treated with the sponge extract from *T*. *aurantium* (Ta), *T*. *citrina* (Tc), *H*. *perlevis* (Hp), *I*. *variabilis* (Iv), *C*. *nucula* (Cn), *A*. *aerophoba* (Aa) and *S*. *spinosulus* (Ss) at the indicated concentrations. After 24 h of incubation at 37°C, 5% CO_2_ astrocytes were subjected to the MTT assay. The results are expressed as percentage of surviving cells over untreated cells. Data are presented as mean ± SD of three different experiments with independent cell populations. The horizontal dashed line, set at 60%, indicates the threshold of cell viability. Concentrations of the crude extracts that yielded cell viability values < 60% of control were considered as toxic doses.

In order to ascertain whether the reduction of MMP-9 and MMP-2 levels, observed in astrocytes after treatment with the different concentrations of sponge extracts, was not due to cytotoxic effects of the extracts on cells, astrocytes were subjected to the MTT assay. As shown in Fig 3 the extracts at the used concentrations did not exert cytotoxic or proliferative effects on astrocytes, suggesting the presence in the aqueous extracts of water-soluble compounds with inhibitory activity towards MMPs.

### In-Gel Inhibition of MMP-2 and MMP-9 activity after incubation with the aqueous extracts from the studied sponges

**Fig 4:**
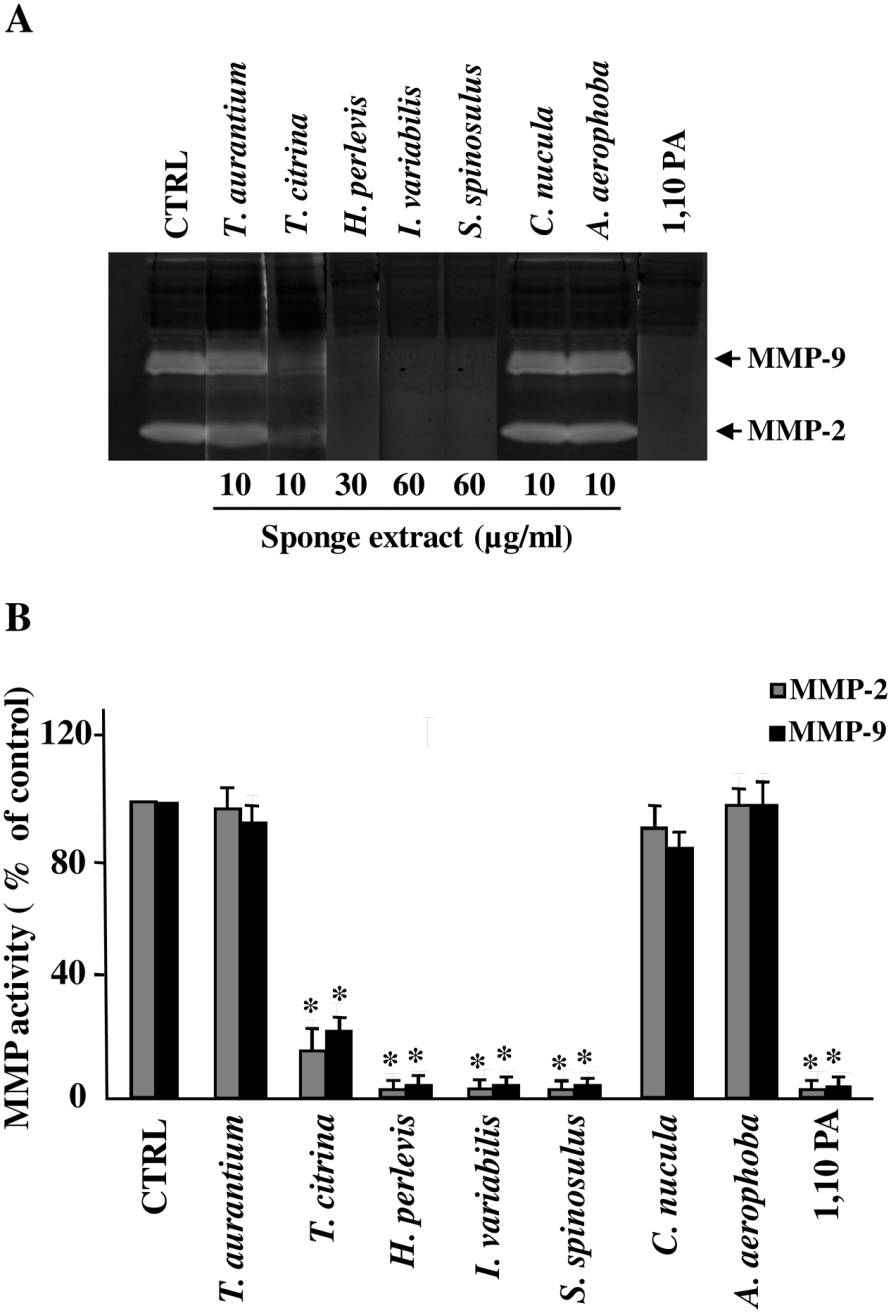
In-gel inhibition of MMP-2 and MMP-9 activity by sponge extracts. MMP-2 and MMP-9 standard were applied to gelatin-zymography. After the electrophoretic run in SDS, gels were cut in lanes and each lane was washed with Triton X-100 and incubated overnight in the incubation medium (50 mMTris; 1% Triton-X-100; 10 mM CaCl_2_; 0.2% NaN_3_; pH 7.5) in the absence or in the presence of the different aqueous extracts at the highest non-cytotoxic concentrations. 1,10 PA was used as a positive control. **A**: Staining and destaining of the gels revealed that *T*. *citrina*, *H*. *perlevis*, *I*. *variabilis* and *S*. *spinosulus* but not *T*. *aurantium*, *C*. *nucula* and *A*. *aerophoba*, were able to inhibit both MMP-2 and MMP-9. **B**: Percentage of MMP-9 and MMP-2 inhibition of repeated analyses, calculated in comparison to negative control (CTRL) represented by the lane incubated in the absence of the sponge extracts. Asterisks represent values statistically different from control (One-way Anova followed by Tukey test; * = p<0.05).

An *in vitro* assay, already descibed in [19], was used to test the ability of the crude extracts from the different sponges to inhibit MMP-9 and MMP-2 activity. Briefly, exogenous MMP-2 and MMP-9 were separated by gelatin-zymography, then each lane of the zymogram was incubated in developing buffer containing the crude extracts from the different sponge at the highest non-cytotoxic concentration. A lane of the zymogram was incubated in the presence of 1,10 phenantrolin (PA), a specific inhibitor of MMPs (positive control). A representative gel is reported in Fig 4A. Percentages of inhibition of MMP-2 and MMP-9 activity of repeated analyses are reported in Fig 4B. As shown, the treatment with PA completely blocked the activity of MMP-2 and MMP-9. Similar results were observed after incubation of gels with the crude extracts from *T*. *citrina*, *H*. *perlevis*, *I*. *variabilis* and *S*. *spinosulus*, indicating that the aqueous extracts from these sponges possibly contain compounds able to inhibit directly the activity of the enzymes.

### Inhibition of MMP-2 and MMP-9 mRNA expression in astrocytes by *T*. *aurantium*, *H*. *perlevis*, *C*. *nucula* and *A*. *aerophoba*

**Fig 5:**
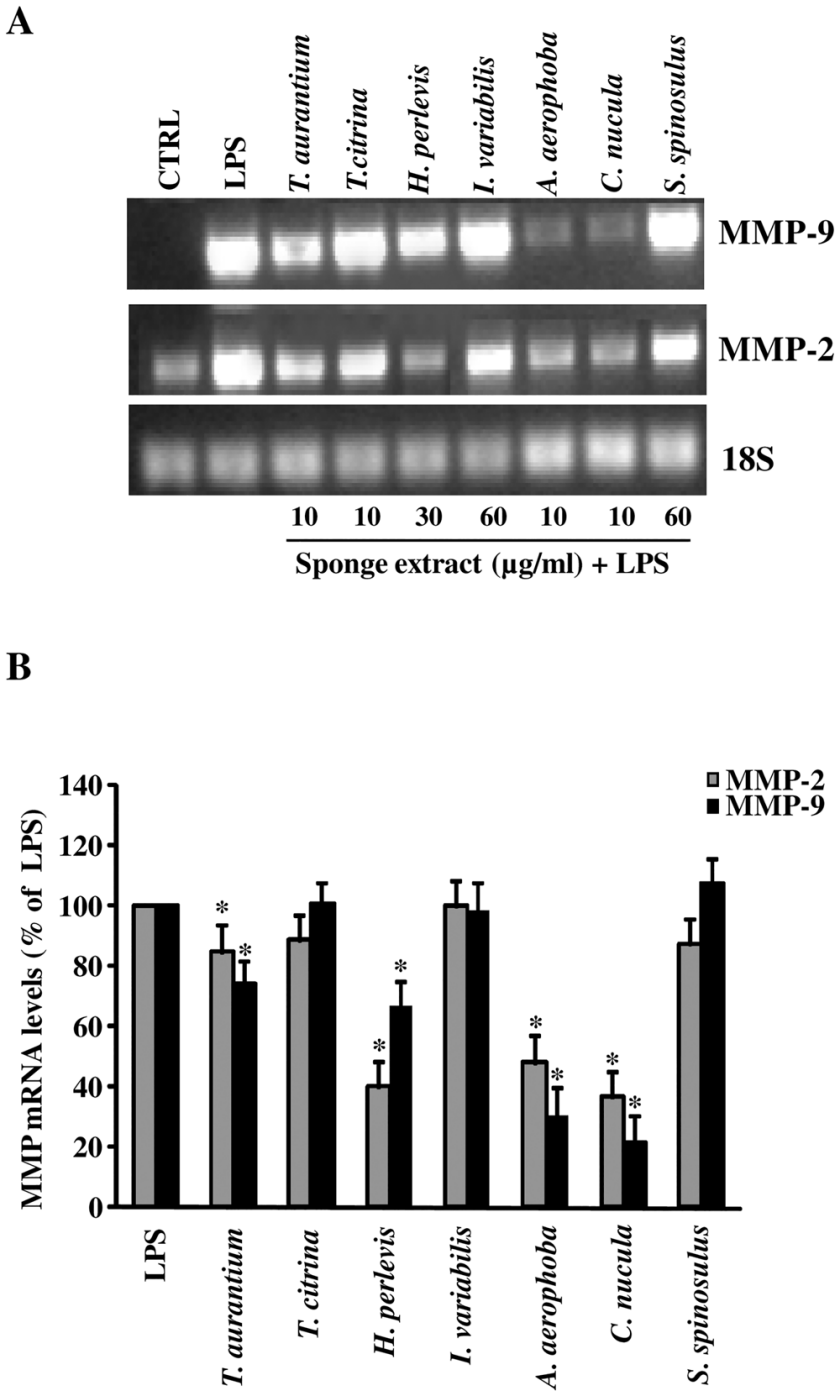
Effect of treatment with the different sponge extracts on MMP-2 and MMP-9 mRNA expression in astrocytes. Primary astrocytes (1×10^5^ cells/ml) were activated with LPS (10 μg/ml) (positive control) and treated with each aqueous extract at the indicated concentrations. Untreated and unstimulated cells represent negative control (CTRL). After 24 h of incubation at 37°C, 5% CO_2_, total RNA was isolated from cells and RNA samples were analyzed by RT-PCR, using the primer pairs specific for MMP-2, MMP-9 and 18S. The products were run on a 1.5% agarose gel containing ethidium bromide. The bands were visualized under UV. Representative results are shown in **A**. Quantitation of the above experiment and two others after scanning densitometry are shown in **B**. Positive control MMP-2 and MMP-9 mRNA were set at 100%, and the treatments with the aqueous extracts represented as the percent of control (mean ±SD). Statistically significant inhibition of MMP mRNA expression in comparison to positive control (LPS) is indicated by asterisks (one way ANOVA followed by Tukey test; * = p<0.05).

As assessed by RT-PCR (Fig 5A), a statistical significant inhibition of the expression of MMP-2 and MMP-9 mRNA was observed in astrocytes activated with LPS and simultaneously treated with the crude extracts from *T*. *aurantium*, *C*. *nucula*, *A*. *aerophoba* and *H*. *perlevis* at the highest non-cytotoxic concentration. By contrast, no inhibition of both MMP-2 and MMP-9 mRNA expression was observed in cells treated with *T*. *citrina*, *I*. *variabilis* and *S*. *spinosulus*. Fig 5B shows the results from multiple experiments with different cell populations after normalization with 18S mRNA, used as an internal control.

### ERK-1/2 is involved in the inhibition of MMP-9 expression by *T*. *aurantium*, *H*. *perlevis*, *C*. *nucula* and *A*. *aerophoba*

**Fig 6:**
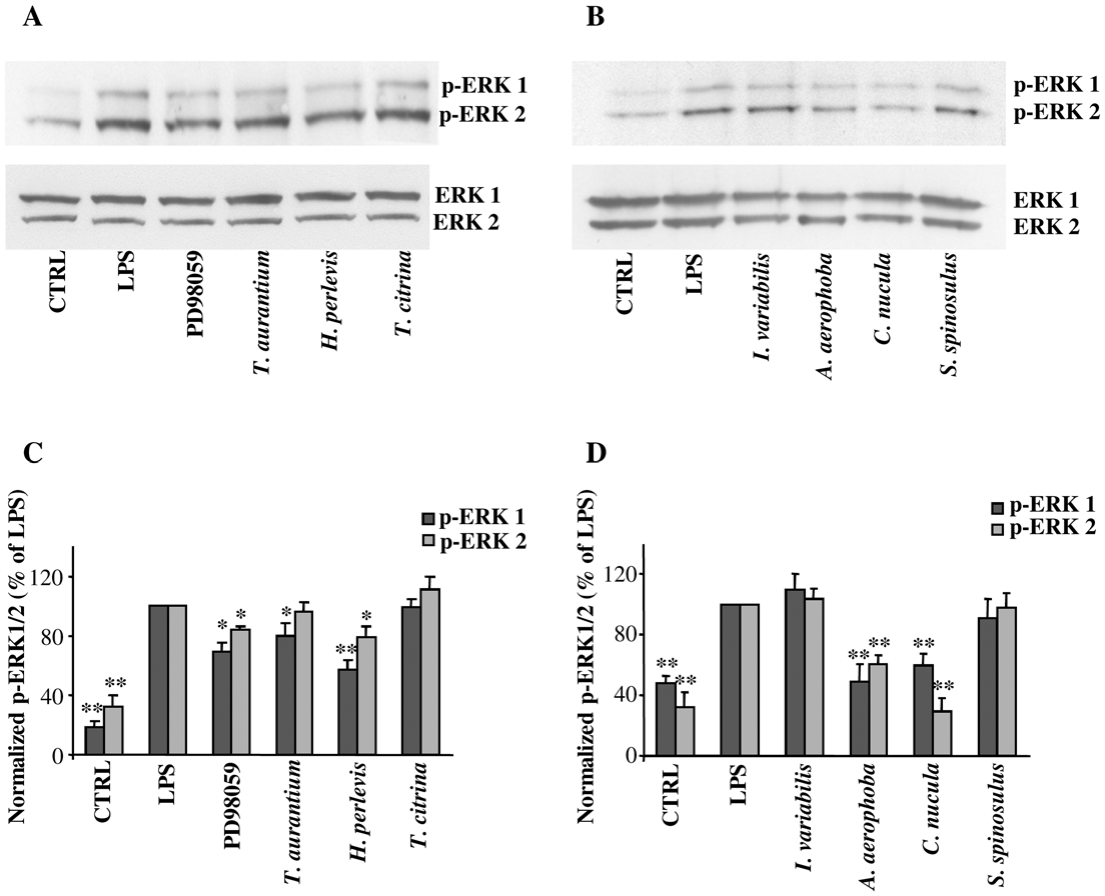
Inhibitory effect of the different sponge extracts on ERK 1/2 signalling pathway in astrocytes. Primary astrocytes (1×10^5^ cells/ml) were pre-treated for 2 h with the crude extracts from *T*. *aurantium* (10 μg/ml), *H*. *perlevis* (30 μg/ml), *T*. *citrina* (10 μg/ml), *I*. *variabilis* (60 μg/ml), *A*. *aerophoba* (10 μg/ml), *C*. *nucula* (10 μg/ml), and *S*. *spinosulus* (60 μg/ml) or with 10 μM of the ERK 1/2 inhibitor PD98059, then activated for 2 h with LPS (10 μg/ml). Untreated and unstimulated cells represent negative control (CTRL). Representative autoradiographic films of Western blotting analysis are reported in **A** and **B**. Histograms in **C** and **D** represent the results, after densitometric scanning of autoradiographic films, normalized as the ratio of phosphorylated to total ERK protein. Data represent means ± SD of three independent experiments. Asterisks represent values statistically different from positive control (LPS-activated astrocytes), which was set at 100% (one-way ANOVA followed by Tukey test; *p < 0.05).

To elucidate the molecular mechanism of inhibition of MMP-9 by sponge extracts we tested their effect on the activation of extracellular-regulated protein kinases (ERK) 1/2, which is the main signaling transduction pathway involved in the regulation of MMP-9 expression in LPS-activated astrocytes [28]. As shown in Fig 6, pretreatment of LPS-activated astrocytes with the extracts from *T*. *aurantium*, *H*. *perlevis*, *A*. *aerophoba* and *C*. *nucula*. significantly reduced phosphorylated ERK (p-ERK) 1/2 levels in a way comparable to that observed in astrocytes pretreated in the same experimental conditions with the ERK inhibitor PD98059. No reduction of p-ERK 1/2 was observed after pretreatment of LPS-activated astrocytes with *T*. *citrina*, *I*. *variabilis* and *S*. *spinosulus*.

### Aqueous extracts from reared specimens of *T*. *aurantium* and *T*. *citrina* inhibit MMP-2 and MMP-9 in LPS-activated astrocytes

**Fig 7:**
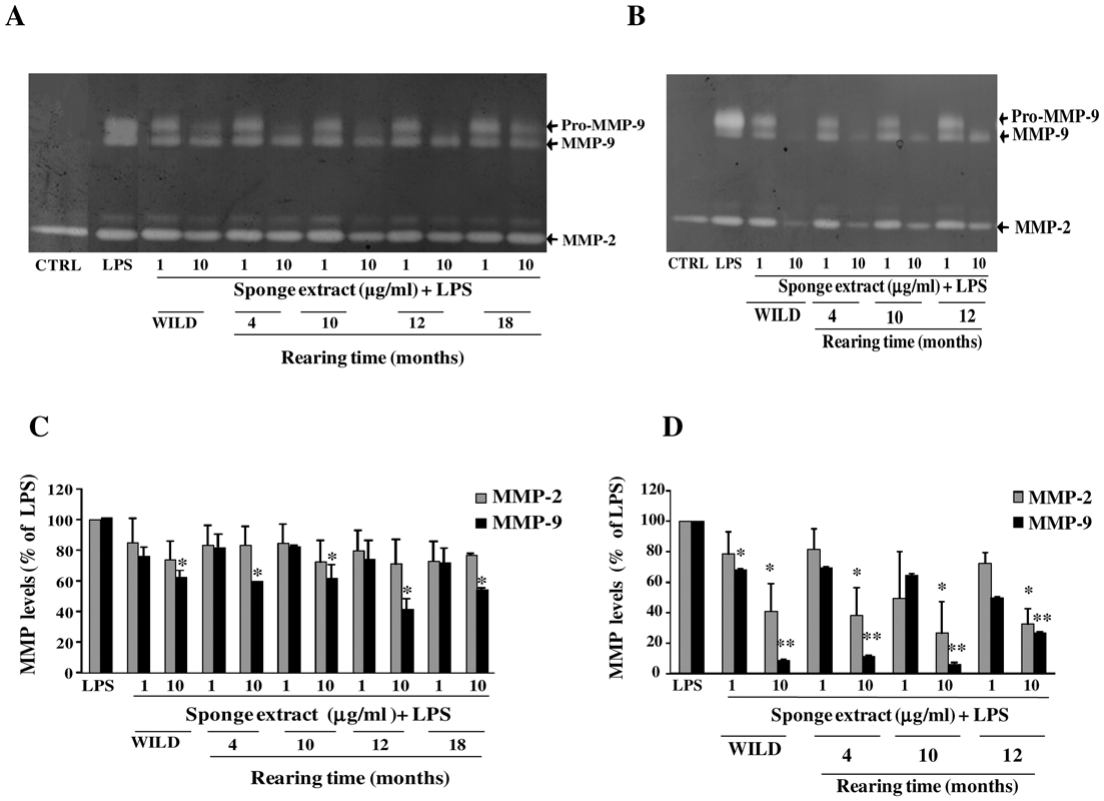
Inhibition of MMP-9 and MMP-2 levels by the sponge extracts from wild and reared specimens of *T*. *aurantium* and *T*. *citrina*. Primary astrocytes (1×10^5^ cells/ml) were activated with LPS (10 μg/ml) and treated with the crude extracts from *T*. *aurantium* and *T*. *citrina* at the indicated concentrations. Untreated and unstimulated cells represent negative control (CTRL). Culture supernatants were harvested after 24 h of incubation and subjected to gelatin-zymography. Representative gels in A and B show the effect of the treatment with the crude extracts from wild and reared specimens of *T*. *aurantium* (A) and *T*. *citrina* (B) at the indicated rearing times. MMP-2 and MMP-9 were identified by their apparent molecular mass of 67 and 92 kDa, respectively, using pre-stained molecular weight markers (Bio Rad). Histograms in C and D represent results, expressed as mean ± SD, after scanning densitometry and computerized analysis of gels from at least three independent experiments with different cell populations. Asterisks represent values statistically different from LPS-treated astrocytes (positive control) (One-way Anova followed by Tukey test; * = p< 0.05; ** = p< 0.01).

To evaluate if aqueous extracts from sponges could retain their biological activities during the cultivation period, specimens of *T*. *aurantium* and *T*. *citrina*, were reared for 4, 10, 12, 18 months andfor 4, 10, 12 months, respectively. Their extracts were then tested for the biological activity against MMP-2 and MMP-9 on LPS-activated astrocytes. As shown in the representative gels reported in Fig 7A and 7B, the treatment of LPS-activated astrocytes with aqueous extracts from wild and reared specimens of *T*. *aurantium*, at the indicated rearing times, inhibited MMP-9 levels (Fig 7A) whereas the aqueous extracts from wild and reared specimens of *T*. *citrina* showed an inhibition of both MMP-2 and MMP-9 levels (Fig 7B). Results from multiple experiments with different cell populations are reported in the histograms in Fig 7C and 7D.

### Inhibition of MMP-9 mRNA expression in LPS-activated astrocytes by aqueous extracts from reared specimens of *T*. *aurantium*

**Fig 8:**
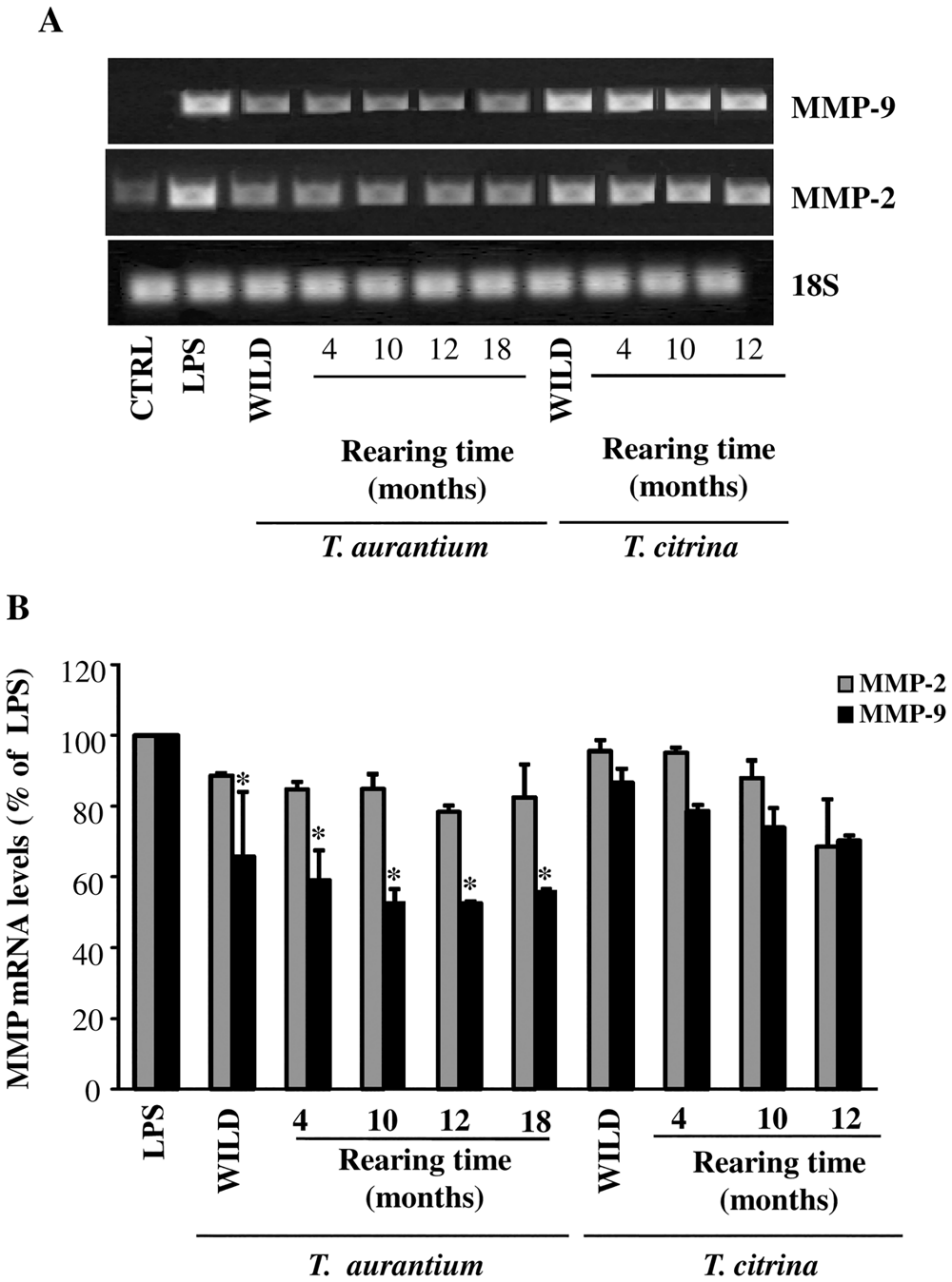
Effect of treatment with the *T*. *aurantium* and *T*. *citrina* sponge extracts from wild and reared specimens on MMP-2 and MMP-9 expression in astrocytes. Primary astrocytes (1×10^5^ cells/ml) were activated with LPS and treated with the crude extracts from *T*. *aurantium* and *T*. *citrina* at the indicated concentrations. Untreated and unstimulated cells represent negative control (CTRL). The isolated RNA samples were analyzed by RT-PCR, using the primer pairs specific for MMP-2, MMP-9 and 18S. The products were run on a 1.5% agarose gel containing ethidium bromide. The bands were visualized under UV. Representative results are shown in A. Quantitation of the above experiment and two others after scanning densitometry are shown in B. Positive control MMP-2 and MMP-9 mRNA were set at 100%, and the treatments with the aqueous extracts represented as the percent of control (mean ±SD). Statistically significant inhibiton of MMP mRNA expression in comparison to positive control (LPS) is indicated by asterisks (one way ANOVA followed by Tukey test; * = p<0.05).

RT-PCR experiments, reported in Fig 8 revealed that the expression of MMP-9 mRNA was significantly inhibited in LPS-activated astrocytes treated with the aqueous extract from wild and reared specimens of *T*. *aurantium*. Conversely, the treatment with the aqueous extracts from wild and reared specimens of *T*. *citrina* did not inhibit the expression of both MMP-2 and MMP-9. In addition, the inhibitory mechanisms exerted by the reared specimens of *T*. *aurantium* and *T*. *citrina* occurred similarly to those observed in their wild conspecifics.

## Discussion

The marine environment is characterized by high biodiversity offering a vast variety of natural products, which could be used as potential drugs against various human diseases [22]. In recent years, marine organisms have been studied extensively for their biochemical responses to evade potential threats present in marine environment. This has led to the idea of exploiting their bioactive compounds to be used as potential inputs in pharmacological applications with the ultimate goal to uplift human health [33]. Among marine organisms, marine sponges, by being the most diversified faunal communities of seas, have significantly contributed in discovering bioactive compounds. Potential drug discovery targets include matrix metalloproteinases (MMPs), and in particular gelatinases A (MMP-2) and B (MMP-9), which are involved at different levels in the pathogenesis of several human diseases [34–36].

In this research aqueous extracts prepared from seven Mediterranean demosponges were tested on cultured astrocytes with the final aim of evaluating the presence of water-soluble bioactive compoundswith anti-MMP activity. The rationale for the choice of astrocytes as cell target comes from different observations: i) they represent an experimental model successfully used in other studies [17,18,31]; ii) these cells have been already tested for the assessment of the non-cytotoxic concentrations of the crude extracts [26]; iii) they play an important role in the pathogenesis of neuroinflammatory and neurodegenerative diseases. In fact, it has been extensively documented that during these pathological events, astrocytes are activated and release cytotoxic and myelinotoxic factors such as MMPs [37].

In our experiments, astrocytes were activated with LPS, a well-known inducer of MMPs [28], and treated with the non-cytotoxic concentrations of the aqueous extracts, as preliminary assessed [26]. Water soluble compounds with anti-MMP-2 and MMP-9 activity were found in the extracts from all the studied sponges. As previously demonstrated, the extracts from the seven demosponges studied present very rich protein patterns [26] it is conceivable to attribute the inhibitory effect on MMPs to water-soluble protein compounds. It should be pointed out that until now, the majority of anti-MMP bioactive compounds isolated from marine sponges are mainly organic molecules [23–25]. Therefore, in order to exclude that the inhibitory effect on MMPs could be due to organic secondary metabolites possibly present in the aqueous extracts, we subjected our samples to a further extraction with an organic solvent and found that the aqueous phase, obtained after the extraction, still presented anti-MMP activity comparable with that of the aqueous extract. In addition, it should be emphasized that the electrophoretic profile of the aqueous phase, recovered after treatment with chloroform, was identical to that of the starting aqueous extract. Therefore, although this finding does not represent a direct evidence, it strengthens the hypothesis that the inhibitory effect of the aqueous extracts on MMP-2 and MMP-9 might be due to a protein compound.

Our findings indicate that the aqueous extracts exert their anti-MMP effect at different level. In particular, the results obtained after the ‘‘in gel zymography” and RT-PCR experiments suggest that the aqueous extracts prepared from the studied sponges inhibit differently MMP activity and expression, possibly dependently to the water-soluble compounds present in each extracts [26]. Specifically, the results obtained after the ‘‘in gel zymography” suggest that water-soluble compounds from *T*. *citrina*, *I*. *variabilis* and *S*. *spinosulus* are able to inhibit directly the activity of both MMP-2 and MMP-9 with an efficiency that is comparable with that of 1,10 PA, a specific inhibitor of MMPs. By contrast, the inhibition of MMP-2 and MMP-9 by *T*. *aurantium*, *C*. *nucula* and *A*. *aerophoba* appears to be directed on the expression of the enzymes. It is noteworthy that *H*. *perlevis* inhibited both the activity and the expression of MMP-2 and MMP-9, suggesting the concomitant presence of water-soluble compounds with different types of anti-MMP activity.

Concerning the ability of some extracts to directly inhibit MMP activity, we can hypothesize that some water-soluble compounds contained in the aqueous extracts may interact with the catalytic site of MMPs exerting an effect similar to that of TIMPs. In this light, a 21 kDa proteinase from the muscle of Atlantic cod, *Gadus morhua*, showing properties similar to human TIMP-2, has been purified. The 21 kDa proteinase was found to inhibit the gelatinolytic activity obtained from a human macrophage cell medium rich in MMP-9 [38]. In this respect, it should be mentioned that the SDS-PAGE analysis of the aqueous extract from *H*. *perlevis* was characterized by the presence of a major band of 21 kDa [26]. Further analyses are in progress to ascertain its possible gelatinolytic activity.

The inhibition of MMP-2 and MMP-9 expression by *T*. *aurantium*, *C*. *nucula* and *A*. *aerophoba* suggests the presence in these extracts of water-soluble compounds possibly able to interfere with the signaling pathways leading to the activation of MMP gene.

To better understand the mechanisms underlying the inhibition of MMPs, we focused our attention on ERK 1/2, which is the main signaling transduction pathway involved in the regulation of MMP-9 expression in LPS-activated astrocytes [28]. As observed, phosphorylated ERK (p-ERK) 1/2 levels were significantly reduced in the same extracts which showed a significant inhibition of MMP-9 gene expression. Conversely, no reduction of p-ERK 1/2 was observed after pretreatment of LPS-activated astrocytes with the sponge extracts which did not show any inhibition of MMP-9 expression. This result suggests that a mechanism by which the aqueous extracts from *T*. *aurantium*, *H*. *perlevis*, *A*. *aerophoba* and *C*. *nucula* inhibit MMP-9 is through the inhibition of MMP-9 gene trascription. In this respect, in a previous research we demonstrated that plant-derived bioactive compounds with antioxidant activity are able to inhibit MMP-9 expression by reducing the activation of ERK signaling pathway [31].

The presence of compounds able to inhibit MMP activity and expression in the aqueous extracts encourages to identify and purify the bioactive protein compounds from the studied sponges. However, sponge tissues usually bear only traces of the compounds of pharmacological interest [39], therefore the use of large amounts of sponge biomass is required for the commercial production of these bioactive compounds [40]. Currently, this “supply problem” still hampers the pharmacological use of many promising metabolites from marine organisms [41]. Sponge aquaculture is one possible method that could supply sufficient and sustainable quantities of sponge metabolites for drug development and manufacture [42,43]. In this respect, our research group have recently developed rearing methodologies for the ex situ cultivation of *T*. *aurantium* and *T*. *citrina* reared for a period of 18 and 12 months, respectively [44].

On these grounds, in this research we also evaluated whether aqueous extracts from reared specimens of *T*. *aurantium* and *T*. *citrina* could maintain their biological activities during the rearing period demonstrated that the extracts from the reared specimens of *T*. *aurantium* were able to inhibit in a dose-dependent manner MMP-9 levels in LPS-activated astrocytes, while those from *T*. *citrina* inhibited dose-dependently levels of both gelatinases. In addition, these inhibitory activities appeared to be maintained throughout the rearing period. Moreover, the mechanisms of inhibition exerted by the reared specimens of *T*. *aurantium* and *T*. *citrina* occurred similarly to those observed in their wild conspecifics.

In our previous research we demonstrated that the protein profile of these reared sponges showed increased levels of two protein bands of 50 and 36 kDa. In particular, the 50 kDa levels were increased in both the species, while the 36 kDa only in *T*. *citrina* [44]. Therefore, since the observed inhibition in the aqueous extracts of the reared specimens of the studied species did not change during the rearing period, we can hypothesize that the inhibition of gelatinases A and B might not be correlated to these induced proteins, but to other water soluble compounds or, more specifically, to other proteins, which are constitutively expressed in these sponges.

On the whole, our results indicate that the aqueous extracts from the selected Mediterranean demosponges possess a variety of water-soluble bioactive compounds, which are able to inhibit MMPs at different levels. In addition, the findings obtained from the treatment of astrocytes with the extracts from the reared sponges, *T*. *aurantium* and *T*. *citrina*, reveal that these sponges maintain the production of compounds with inhibitory activities on MMP-2 and MMP-9 during the rearing period. In conclusion, the results of this research indicate that the water-soluble compounds present in the aqueous extracts of the studied sponges might have possible pharmacological applications. In addition, the biological activities in the extracts from the reared sponges encourage sponge aquaculture as a valid option to supply sponge biomass for drug development on a large scale.

## References

[1] Visse R, Nagase R (2003) Matrix metalloproteinases and tissue inhibitors of metalloproteinases. Circ Res 92: 827–839. 12730128 10.1161/01.RES.0000070112.80711.3D

[2] Allan JA, Docherty AJ, Barker PJ, Huskisson NS, Reynolds JJ, et al (1995) Binding of gelatinases A and B to type-I collagen and other matrix components. Biochem J 309: 299–306. 7619071 10.1042/bj3090299 PMC1135833

[3] Sang QX, Jin YH, Newcomer RG, Monroe SC, Fang XX, et al (2006) Matrix metalloproteinase inhibitors as prospective agents for the prevention and treatment of cardiovascular and neoplastic diseases. Curr Top Med Chem 6: 289–316. 16611144 10.2174/156802606776287045

[4] Egeblad M, Werb Z (2002) New functions for the matrix metalloproteinase in cancer progression. Nat Rev Cancer 2: 161–174. 11990853 10.1038/nrc745

[5] Mastroianni CM, Liuzzi GM (2007) Matrix metalloproteinase dysregulation in HIV infection: implications for therapeutic strategies. Trends Mol Med 13: 449–459. 18029231 10.1016/j.molmed.2007.09.001

[6] Yong VW, Power C, Forsyth P, Edwards DR (2001) Metalloproteinases in biology and pathology of the nervous system. Nat Rev Neurosci 2: 502–511. 11433375 10.1038/35081571 PMC7097548

[7] Campbell IL, Pagenstecher A (1999) Matrix metalloproteinases and their inhibitors in the nervous system: the good, the bad and the enigmatic. Trends Neurosci 22: 285–287. 10484748 10.1016/s0166-2236(99)01430-7

[8] Rosenberg GA (2002) Matrix metalloproteinases in neuroinflammation. Glia 39: 279–291. 12203394 10.1002/glia.10108

[9] Coussens LM, Fingleton B, Matrisian LM (2002) Matrix metalloproteinase inhibitors and cancer: trials and tribulations. Science 295: 2387–2392. 11923519 10.1126/science.1067100

[10] Overall CM, Lopez-Otin C (2002) Strategies for MMP inhibition in cancer: innovations for the post-trial era. Nat Rev Cancer 2: 657–672. 12209155 10.1038/nrc884

[11] Hu JL, Van den Steen PE, Sang QX, Opdenakker G (2007) Matrix metalloproteinase inhibitors as therapy for inflammatory and vascular diseases. Nat Rev Drug Discov 6: 480–498. 17541420 10.1038/nrd2308

[12] Pavlaki M, Zucker S (2003) Matrix metalloproteinase inhibitors (MMPIs): the beginning of phase I or the termination of phase III clinical trials. Cancer Metastasis Rev 22: 177–203. 12784996 10.1023/a:1023047431869

[13] Skiles JW, Gonnella NC, Jeng AY (2004) The design, structure, and clinical update of small molecular weight matrix metalloproteinase inhibitors. Curr Med Chem 11: 2911–2977. 15544483 10.2174/0929867043364018

[14] Liuzzi GM, Latronico T, Fasano A, Carlone G, Riccio P (2004) Interferon-beta inhibits the expression of metalloproteinases in rat glial cell culture: implications for multiple sclerosis pathogenesis and treatment. Mult Scler 10: 290–297. 15222694 10.1191/1352458504ms1016oa

[15] Liuzzi GM, Mastroianni CM, Latronico T, Mengoni F, Fasano A, et al (2004) Anti-HIV drugs decrease the expression of matrix metalloproteinases in astrocytes and microglia. Brain 127: 398–407. 14662518 10.1093/brain/awh049

[16] Latronico T, Liuzzi GM, Riccio P, Lichtner M, Mengoni F, et al (2007) Antiretroviral therapy inhibits matrix metalloproteinase-9 from blood mononuclear cells of HIV-infected patients. AIDS 21: 677–684. 17413688 10.1097/QAD.0b013e328018751d

[17] Gramegna P, Latronico T, Branà MT, Di Bari G, Mengoni F, et al (2011) In vitro downregulation of matrix metalloproteinase-9 in rat glial cells by CCR5 antagonist maraviroc: Therapeutic Implication for HIV Brain Infection. PLoS ONE 6: e28499 10.1371/journal.pone.0028499 22174822 PMC3234279

[18] Liuzzi GM, Latronico T, Rossano R, Viggiani S, Fasano A, et al (2007) Inhibitory effect of polyunsaturated fatty acids on MMP-9 release from microglial cells-implications for complementary multiple sclerosis treatment. Neurochem Res 32: 2184–2193. 17624613 10.1007/s11064-007-9415-9

[19] Liuzzi GM, Latronico T, Branà MT, Gramegna P, Coniglio MG, et al (2011) Structure-dependent inhibition of gelatinases by dietary antioxidants in rat astrocytes and sera of multiple sclerosis patients. Neurochem Res 36: 517–527. 10.1007/s11064-010-0373-2 21207142

[20] Zhang C, Kim SK (2009) Matrix metalloproteinase inhibitors (MMPIs) from marine natural products: the current situation and future prospects. Mar Drugs 7: 71–84. 10.3390/md7020071 19597572 PMC2707034

[21] Blunt JW, Copp BR, Keyzers RA, Munro MH, Prinsep MR (2014) Marine natural products. Nat Prod Rep 31: 160–258. 10.1039/c3np70117d 24389707

[22] Leal MC, Puga J, Serôdio J, Gomes NCM, Calado R (2012) Trends in the Discovery of New Marine Natural Products from Invertebrates over the Last Two Decades—Where and What Are We Bioprospecting? PLoS ONE 7: e30580 10.1371/journal.pone.0030580 22276216 PMC3262841

[23] Fujita M, Nakao Y, Matsunaga S, Seiki M, Itoh Y, et al (2003) Ageladine A: an antiangiogenicmatrixmetalloproteinase inhibitor from the marine sponge *Agelas nakamurai* . J Am Chem Soc 125: 15700–15701. 14677933 10.1021/ja038025w

[24] Fujita M, Nakao Y, Matsunaga S, Seiki M, Itoh Y, et al (2001) Ancorinosides B-D, inhibitors of membrane type 1 matrix metalloproteinase (MT1-MMP), from the marine sponge *Penares sollasi* Thiele. Tetrahedron 57: 1229–1234.

[25] Rodríguez-Nieto S, González-Iriarte M, Carmona R, Muñoz-Chápuli R, Medina MA, et al (2002) Antiangiogenic activity of aeroplysinin-1, a brominated compound isolated from a marine sponge. Faseb J 16: 261–263. 11772945 10.1096/fj.01-0427fje

[26] Di Bari G, Gentile E, Latronico T, Corriero G, Fasano A, et al (2014) Comparative analysis of protein profiles of aqueous extracts from marine sponges and assessment of cytotoxicity on different mammalian cell types. Environ Tox Pharm 38: 1007–1015. 10.1016/j.etap.2014.10.021 25461562

[27] Latronico T, Branà MT, Gramegna P, Fasano A, Di Bari G, et al (2013) Inhibition of myelin-cleaving proteolytic activities by interferon-beta in rat astrocyte cultures. Comparative analysis between gelatinases and calpain-II. PLoS ONE 8: e49656 10.1371/journal.pone.0049656 23390485 PMC3563665

[28] Lee WJ, Shin CY, Yoo BK, Ryu JR, Choi EY, et al (2003) Induction of matrix metalloproteinase-9 (MMP-9) in lipopolysaccharide-stimulated primary astrocytes is mediated by extracellular signal-regulated protein kinase 1/2 (Erk1/2). Glia 41: 15–24. 12465042 10.1002/glia.10131

[29] Cardone F, Gaino E, Corriero G (2010) The budding process in *Tethya citrina* Sara & Melone (Porifera, Demospongiae) and the incidence of post-buds in sponge population maintenance. J Exp Mar Biol Ecol 389: 93–100.

[30] Bradford MA (1976) Rapid and sensitive method for the quantitation of microgram quantities of protein utilizing the principle of protein-dye binding. Anal Biochem 72: 248–254. 942051 10.1016/0003-2697(76)90527-3

[31] Latronico T, Branà MT, Merra E, Fasano A, Di Bari G, et al (2013) Impact of manganese neurotoxicity on MMP-9 production and superoxide dismutase activity in rat primary astrocytes. Effect of resveratrol and therapeutical implications for the treatment of CNS diseases.Toxicol Sci 135: 218–228. 10.1093/toxsci/kft146 23811825

[32] Heussen C, Dowdle EB (1980) Electrophoretic analysis of plasminogen activators in polyacrylamide gels containing sodium dodecyl sulfate and copolymerized substrates. Anal Biochem 102: 196–202. 7188842 10.1016/0003-2697(80)90338-3

[33] Sipkema D, Franssen MC, Osinga R, Tramper J, Wijffels RH (2005) Marine sponges as pharmacy. Mar Biotechnol 7: 142–162. 15776313 10.1007/s10126-004-0405-5 PMC7087563

[34] Rosenberg GA (2009) Matrix metalloproteinases and their multiple roles in neurodegenerative diseases. Lancet Neurol 8: 205–216. 10.1016/S1474-4422(09)70016-X 19161911

[35] Malemud CJ (2006) Matrix metalloproteinases (MMPs) in health and disease: an overview. Front Biosci 11: 1696–1701. 16368548 10.2741/1915

[36] Mannello F (2006) Natural bio-drugs as matrix metalloproteinase inhibitors: new perspectives on the horizon? Recent Pat Anticancer Drug Discov 1: 91–103. 18221029 10.2174/157489206775246421

[37] Bélanger M, Magistretti PJ (2009) The role of astroglia in neuroprotection. Dialogues Clin Neurosci 11: 281–295. 19877496 10.31887/DCNS.2009.11.3/mbelanger PMC3181926

[38] Lødemel JB, Egge-Jacobsen W, Olsen RL (2004) Detection of TIMP-2-like protein in Atlantic cod (*Gadus morhua*) muscle using two-dimensional real-time reverse zymography. Comp Biochem Physiol 139: 253–259. 15465672 10.1016/j.cbpc.2004.08.004

[39] Koopmans M, Martens D, Wijffels RH (2009) Towards Commercial Production of Sponge Medicines. Mar Drugs 7: 787–802. 10.3390/md7040787 20098610 PMC2810229

[40] Munro MHG, Blunt JW, Dumdei EJ, Hickford SH, Lill RE, et al (1999) The discovery and development of marine compounds with pharmaceutical potential. J Biotechnol 70: 15–25. 10412202 10.1016/s0168-1656(99)00052-8

[41] Newman DJ, Cragg GM (2004) Marine Natural Products and Related Compounds in Clinical and Advanced Preclinical Trials. J Nat Prod 67: 1216–1238. 15332835 10.1021/np040031y

[42] Duckworth AR, Battershill CN (2003) Developing farming structures for production of biologically active sponge metabolites. Aquaculture 217: 139–156.

[43] Duckworth AR, Battershill CN (2003) Sponge aquaculture for the production of biologically active metabolites: The influence of farming protocols and environment. Aquaculture 221: 311–329.

[44] Di Bari G, Cardone F, Gaino E, Liuzzi GM, Nonnis Marzano C, et al (2015) Biological variations in a long-term ex situ cultivation: a Mediterranean demosponge as model system. Mediterr Mar Science 16: 73–81.

